# Endogenous Glucocorticoid Metabolism in Bone: Friend or Foe

**DOI:** 10.3389/fendo.2021.733611

**Published:** 2021-08-27

**Authors:** Claire S. Martin, Mark S. Cooper, Rowan S. Hardy

**Affiliations:** ^1^Institute of Metabolism and Systems Research, University of Birmingham, Birmingham, United Kingdom; ^2^Australian and New Zealand Army Corps (ANZAC) Research Institute, University of Sydney, Sydney, NSW, Australia; ^3^Arthritis Research United Kingdom (UK) Career Development Fellow, University of Birmingham, Birmingham, United Kingdom; ^4^Institute of Clinical Sciences, University of Birmingham, Birmingham, United Kingdom

**Keywords:** glucocorticoid, bone, 11beta-hydroxysteroid dehydrogenase, osteoclast, osteoblast, osteoporosis, chronic inflammation

## Abstract

The role of tissue specific metabolism of endogenous glucocorticoids (GCs) in the pathogenesis of human disease has been a field of intense interest over the last 20 years, fuelling clinical trials of metabolism inhibitors in the treatment of an array of metabolic diseases. Localised pre-receptor metabolism of endogenous and therapeutic GCs by the 11β-hydroxysteroid dehydrogenase (11β-HSD) enzymes (which interconvert endogenous GCs between their inactive and active forms) are increasingly recognised as being critical in mediating both their positive and negative actions on bone homeostasis. In this review we explore the roles of endogenous and therapeutic GC metabolism by the 11β-HSD enzymes in the context of bone metabolism and bone cell function, and consider future strategies aimed at modulating this system in order to manage and treat various bone diseases.

## Introduction to Pre-Receptor Glucocorticoid Metabolism

The role of tissue specific metabolism of endogenous glucocorticoids (GCs) in the pathogenesis of human disease has been a field of intense interest over the last 20 years. This has fuelled clinical trials of chemical inhibitors aiming to prevent metabolic side effects associated with corticosteroid excess, such as insulin resistance, cardiovascular disease and hypertension (1–8). Several enzymes, but most prominently 11β-hydroxysteroid dehydrogenase (11β-HSD) type 1 and 2 play a critical role in regulating peripheral exposure to GCs within tissues *via* their pre-receptor enzyme activity (9, 10). To date, therapeutic interventions have primarily focussed on the GC activating enzyme 11β-HSD1 based on its expression in tissues that are themselves targets for GCs such as bone, where therapeutic GCs drive a rapid and sustained reduction in bone formation and increased risk of fracture (11, 12). Here, global transgenic deletion of 11β-HSD1 in murine models of corticosterone excess have protected animals from deleterious side effects, reinforcing the potential clinical utility of pharmacological inhibition (13, 14). However, the role of corticosteroid activation by 11β-HSD1 selectively within bone, and the cell populations that regulate bone metabolism remain cell and context dependant, and the value of selective 11β-HSD1 inhibitors in clinical practice is unclear. However, given the deleterious impact of therapeutic and endogenous corticosterone excess on bone metabolism, a greater understanding of the role of 11β-HSD1 in bone remains paramount (15). This review examines the latest literature relating to both the role of 11β-HSD1 in bone cells and its regulation of bone metabolism, and further explores the value of therapeutic 11β-HSD1 inhibition to treat osteoporosis.

## Glucocorticoid Metabolism by the 11β-HSD1 and 11β-HSD2 Enzymes

**Figure 1:**
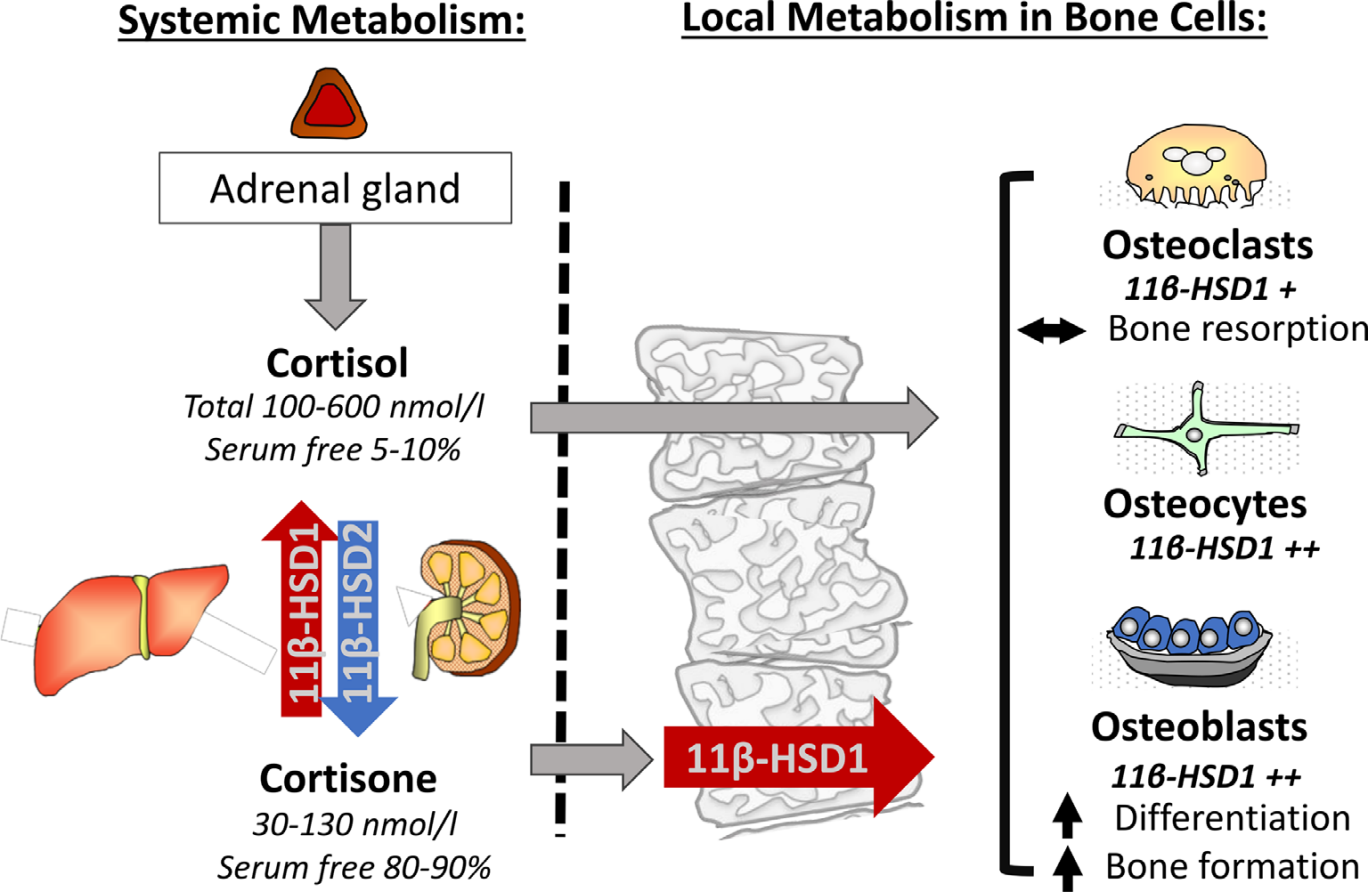
The adrenal corticosteroids cortisol and cortisone achieve a circulating equilibrium predominantly through their renal inactivation by 11β-HSD2 and reactivation by hepatic 11β-HSD1. Both active and inactive serum free corticosteroids are able to enter bone cells. Here 11β-HSD1 can further activate and amplify the actions of corticosteroids through the conversion of cortisone to cortisol where, in combination with serum free cortisol, it influences basal bone metabolism in osteoclasts, osteocytes and osteoblasts.

The 11β-HSDs are intracellular enzymes that interconvert endogenous GCs between their inactive and active forms (**Figure 1**). There are two 11β-HSD enzymes. 11β-HSD1, in the presence of its cofactor nicotinamide adenine dinucleotide phosphate (NADPH), primarily converts the inactive adrenal corticosteroid cortisone to its active counterpart cortisol *via* its oxoreductase activity (converting a ketone on the 11 position of ring C to a hydroxyl group) and conferring increased affinity for the glucocorticoid receptor (GR). This promotes downstream GR signalling (16). In contrast, 11β-HSD2, in the presence of its cofactor nicotinamide adenine dinucleotide (NAD), potently inactivates cortisol to cortisone *via* its dehydrogenase activity, protecting mineralocorticoid receptors in responsive tissues such as kidney, colon and placenta from inappropriate activation by cortisol (17, 18). Final metabolism and urinary clearance of cortisol and cortisone occurs following their metabolism by 5α and 5β-reductases. This, in combination with 3α-hydroxysteroid dehydrogenase activity yields tetrahydrocortisone (THE)/tetrahydrocortisol (THF) and allo THF. The ratio of THF to THE metabolites excreted in the urine can be utilised as a surrogate measure of systemic 11β-HSD1 activity (2). The 11β-HSD enzymes also metabolise several synthetic GCs with 11β-HSD1 activating GCs such as prednisone and 11β-HSD2 inactivating hydrocortisone and prednisolone. However, other synthetic steroids such dexamethasone and methylprednisolone are resistant to metabolism by the 11β-HSD enzymes due to fluorine and methyl group substitutions that significantly reduce metabolic clearance and increase half-life (19, 20). Whilst 11β-HSD2 clearly plays a central role in mineralocorticoid responsive tissues and in determining the circulating cortisol/cortisone ratio, its basal expression outside of these tissues is limited and its importance is less clear. Consequently, this review will primarily focus on the roles of GC metabolism by 11β-HSD1 in the context of bone metabolism and bone cell function.

## Systemic Endocrine Metabolism *Versus* Local Autocrine Metabolism

The roles of 11β-HSD1 span ‘endocrine’ regulation of circulating corticosteroid availability and systemic GC exposure, and the fine-tuning of local tissue and cell specific exposure *via* its ‘autocrine’ activation of cortisol, independently of circulating cortisol. The regulation of systemic endocrine cortisol activation is primarily determined by hepatic 11β-HSD1, which is constitutively and highly expressed within the liver (21, 22). In contrast, the regulation of 11β-HSD1 in tissues, such as adipose, muscle, bone and within sites of inflammation is dynamically regulated in a highly cell and context specific fashion (23–28). Whilst 11β-HSD1 within these tissues also influences circulating endocrine metabolism (albeit to a much lesser extent than hepatic 11β-HSD1), the overwhelming role of 11β-HSD1 in this context is mediated through its autocrine influence on local cortisol exposure independently of circulating cortisol levels. The hypothalamic pituitary adrenal (HPA) axis determines both ultradian and circadian regulation of systemic cortisol levels, with stressors such as inflammation activating production of cortisol by the adrenal gland and negative feedback from GCs suppressing this. The expression of 11β-HSD1 in the suprachiasmatic nucleus, or the “biological clock of the brain”, and the hypothalamus imply a role for this enzyme in circadian regulation of the HPA axis by the negative feedback of active GCs (29). Additionally, 11β-HSD1 in the hippocampus, visceral adipose tissue and subcutaneous adipose tissue has been identified to exhibit circadian variation in gene expression and enzyme activity (30, 31). Whilst circadian and ultradian rhythm have not been shown to be substantially affected in murine models with transgenic 11β-HSD1 deletion, its precise role in central circadian regulation in response to stress and inflammation remain poorly defined. Given its potent inflammatory regulation within myeloid and mesenchymal derived population, it has been hypothesised that chronic activation of 11β-HSD1 may represent a causative factor in the dysregulation of the HPA axis in inflammatory disease (32). Systemic GC activation by hepatic 11β-HSD1 and inactivation by renal 11β-HSD2 play a central role in establishing the circulating ratio of cortisol to cortisone, with levels of active cortisol (typically ranging from 100-600 nmol/l) being 5-6 times higher than for cortisone (30-130 nmol/l) (27). Whilst this circulating ratio helps determine endocrine GC signalling, its function extends to the provision of cortisone as a substrate for local 11β-HSD1 cortisol activation in peripheral tissues. For both aspects of circulating corticosteroid action, one further factor, corticosteroid-binding globulin (CBG) should be considered. Here, approximately 90-95% of total circulating cortisol is sequestered by CBG, and to a lesser extent serum albumin, preventing cell entry and GR mediated cell signalling (33–35). Its relevance to local 11β-HSD1 signalling arises due to a greatly reduced affinity of CBG (roughly 10-fold less) for the inactive corticosteroid, cortisone (33, 36, 37). Consequently, whilst total cortisone circulates at a 5-6-fold lower concentration than total cortisol, serum free cortisone levels available for local autocrine amplification by 11β-HSD1 can match or exceed that of serum free cortisol (**Figure 1**). To appreciate the roles that 11β-HSD1 plays in utilising and activating this abundant pool of circulating serum free cortisone within peripheral tissues such as bone, one must first delineate its cellular expression and distribution within bone itself.

## The Role of 11β-HSD1 in Bone Cells and Bone Health in Normal Physiology

### The Role of Endogenous GCs in Bone Cell Differentiation and Activity

Despite their well-known deleterious effects on bone at therapeutic doses, endogenous GCs play key roles in the formation and maintenance of bone under homeostatic conditions. Continued GC signalling is required for maintenance of adequate bone mass, as seen in models with targeted deletion of GR in osteoblast progenitors or ectopic expression of 11β-HSD2 in mature osteoblasts and osteocytes which exhibit reductions in bone density (38–40). At the individual cell level, GCs promote differentiation of osteoblasts from mesenchymal cells *via* the Wnt/β-catenin pathway. Ectopic expression of the 11β-HSD2 gene in mature osteoblasts *via* the Col2.3 promoter or abrogation of Wnt signalling instead induces adipocyte lineage commitment (41–43). GCs have also been identified to drive differentiation of osteoclasts from mesenchymal precursors and enhance the bone resorption activity of mature osteoclasts (44–46). As well as direct effects on bone cells, GCs influence bone metabolism *via* paracrine signalling. GC stimulation of osteoblasts and osteocytes induces production of receptor activator of nuclear factor kappa-B ligand (RANKL) while suppressing expression of the RANKL decoy receptor osteoprotegerin (OPG), resulting in survival and activation of local osteoclasts (47–50). Within normal physiological conditions, this GC-mediated regulation of bone metabolism functions under strict homeostatic control to carefully balance anabolic and catabolic effects on target cells. Whilst GCs have stimulatory effects on osteoblasts at low doses, they are inhibitory at higher doses, where they instead promote apoptosis of osteoblasts (51, 52). Similarly, GC regulation of mature osteoblast function *via* expression of Wnt proteins functions in a dose-dependent ‘biphasic’ manner (53). Local activation of GCs by the enzyme 11β-HSD1, at both the autocrine and paracrine levels, helps determine available GC for normal physiological responses, as well as potential roles in states of inflammation and GC excess. The functional impact of 11β-HSD1 in bone metabolism has been demonstrated in normal physiology and states of GC excess (54, 55). Systemic 11β-HSD1 activity, and the inactive 11β-HSD1 substrate cortisone, negatively correlated with measures of anabolic bone formation by osteoblasts in cross sectional population studies and was shown to increase with ageing. Furthermore, enzymatic activity of 11β-HSD1 in *ex vivo* grown bone cells was found to increase with donor age (56). These changes were independent of circulating ‘endocrine’ cortisol, suggesting that 11β-HSD1 within cells such as osteoblasts underpinned these observations. Whilst osteoblasts were highlighted as a primary anti-anabolic target of GC metabolism by 11β-HSD1, its dynamic regulation of expression across multiple cell types, including osteocytes, osteoclasts and endothelial cells, hint at a highly cell and context specific role of 11β-HSD1 in bone metabolism *in vivo*.

### The Role of 11β-HSD1 in Osteoblasts and Osteocytes in Normal Physiology

Osteoblasts were initially shown to possess the highest levels of 11β-HSD1 in bone by immunohistochemistry and *in situ* hybridisation (57, 58). Whilst patient studies had reported a potential anti-anabolic role of 11β-HSD1 with ageing, corticosteroid excess and post menopause, initial *in vitro* studies revealed that the upregulation of 11β-HSD1 in immature osteoblast precursors facilitated their differentiation into osteoid producing osteoblasts (59). These findings fit with the well characterised *in vitro* actions of GCs on cultures of osteoblasts, where they stimulate differentiation *via* regulation of specific growth factors and Wnt signalling molecules (41–43). Consequently, these data revealed a potential anabolic role for autocrine cortisol production by 11β-HSD1 in osteoblasts in normal physiology. Murine models in the DBA-1 strain in which GC activation was blocked selectively within osteoblasts supported this hypothesis. Specifically, the overexpression of 11β-HSD2 selectively within osteoblasts, under control of the 2.3Kb Col1a1 promoter, resulted in potent cortisol inactivation to offset endogenous 11β-HSD1 activity (38, 60–62). These animals presented with reduced vertebral bone density and attenuated cranial ossification and reduced periosteal circumference indicating that 11β-HSD1 was required for normal osteoblastic bone formation. Interestingly, similar experiments in the C57BL/6 strain using the osteocalcin promoter, and C57BL/6 animals with a global deletion of 11β-HSD1 failed to reproduce these findings raising doubts as to this explanation (13, 63). Here, the selection of the mouse strain itself may explain this discrepancy, since C57BL/6 mice are reported to have reduced responsiveness to the action of GCs on anabolic bone formation (64). Whilst this can be overcome at higher exogenous corticosteroid doses, these findings suggest that the C57BL/6 strain may not be suited to examining the actions of endogenous GCs in this setting (13). Ultimately, osteoblast targeted deletion of 11β-HSD1 in an appropriate murine strain is still required to address these questions. The anabolic role of 11β-HSD1 in osteoblast differentiation has been less clear in human population studies. This may reflect the respective cohorts examined, where factors such as ageing and exogenous corticosteroid administration may see 11β-HSD1 move from an anabolic role to one mediating corticosteroid excess and facilitating osteoblast autophagy and apoptosis (65, 66). Certainly, in this context measures of 11β-HSD1 activity negatively correlate with markers of bone formation such as osteocalcin and procollagen type 1 amino-terminal propeptide (P1NP) (54–56). It may be that the anabolic actions of 11β-HSD1 may be more apparent in a younger population. Certainly, trials using therapeutic inhibitors of 11β-HSD1 have yet to identify significant changes on bone metabolism in phase II trials (1–8). However, their penetrance within bone has yet to be validated, and so their role in osteoblast differentiation *in vivo* cannot yet be ruled out in humans. Ultimately, more targeted approaches are still required to examine the anabolic roles of 11β-HSD1 *in vivo* in regulating bone metabolism in normal physiology. However, whether therapeutic inhibition of 11β-HSD1 is able to prevent the reported anti-anabolic effects of GCs in osteoblasts in ageing, or post menopause has yet to be determined.

### The Role of 11β-HSD1 in Osteoclasts in Normal Physiology

Analysis of human bone samples confirmed that osteoclasts also express functional 11β-HSD1, but, similar to other bone cell populations, not 11β-HSD2. To assess the functional importance of pre-receptor metabolism of GCs by 11β-HSD1 healthy volunteers were treated with the nonspecific 11β-HSD inhibitor carbenoxolone. After 7 days of treatment, urinary analysis showed normal levels of bone formation markers C- and N-terminal pro-peptides of type I collagen (P1CP and P1NP, respectively) but decreased pyridinoline and deoxypyridinoline, metabolites of bone degraded by osteoclasts. This finding implies a role for local activation of GCs in homeostatic bone resorption (67). In support of this, the selective 11β-HSD1 inhibitor KR-67500, which was found to ameliorate disease in a mouse model of type 2 diabetes, promoted osteoblast maturation of C2C12 cells while blocking RANKL-induced differentiation of murine bone marrow derived macrophages to osteoclasts. Specifically 11β-HSD1 inhibition was found to decrease the genes *Ctsk*, *Fos*, *Nfatc1* and *Dcstamp*, which are required for cellular fusion and multinucleation and bone resorption (68). Despite these findings, clinical trials of 11β-HSD1 inhibitors in diseases such as diabetes, metabolic syndrome, Alzheimer’s, and glaucoma appear to have a favourable safety profile in terms of bone health, with minimal adverse effects reported (1, 3, 69). A trial of 11β-HSD1 inhibition in idiopathic intracranial hypertension specifically assessed serum levels of osteocalcin and sclerostin and measured bone mineral content by dual-energy X-ray absorptiometry (DXA) and found no differences in bone metabolism with treatment (8). Blockade of 11β-HSD1 activation of GCs by ectopic expression of the 11β-HSD2 gene in osteoclasts did not drive any negative skeletal phenotype in mice, with Jia et al. reporting normal bone development, mass and cell numbers (70). Similarly, the 11β-HSD1 knock-out mouse does not appear to have any defects in bone development or structure including under conditions of ageing (13, 71). This suggests that though 11β-HSD1 may play a role in increasing local GC levels for osteoclast differentiation and function, there is inbuilt redundancy in GC regulation of bone metabolism in normal physiological conditions. There is therefore no clear role for 11β-HSD1 in mediating bone homeostasis *via* modulation of endogenous GCs in osteoblasts and osteoclasts, at least under healthy steady state conditions. However, ageing has been shown to increase levels of circulating GCs as well as expression of 11β-HSD1, where dysregulated bone homeostasis frequently presents as conditions such as osteoporosis (72).

Together, whilst these studies suggest that 11β-HSD1 inhibition may have some limited actions on osteoblast and osteoclast maturation and function, its impact on total bone metabolism in normal physiology appear negligible. However, further examination of their effects on bone metabolism across factors such as ageing warrant further investigation.

## 11β-HSD1 Mediates the Anti-Anabolic Actions of Glucocorticoid Excess in Bone

Therapeutic GCs are widely utilised in the treatment of both acute and chronic inflammation, and they are the second most common cause of secondary osteoporosis and increased fracture risk (73, 74). Rapid bone loss over several months occurs after initiation of GCs, followed by a more gradual loss with long term use (11). The dose and duration are significant factors in determining the rate and severity of glucocorticoid-induced osteoporosis (GIOP), and suppression of bone formation (75). The underlying pathology of disease for which GCs is utilised invariably influences this process, with chronic inflammation being a well-described driver of systemic bone loss (75). However, the independent action of glucocorticoids in the absence of inflammation has been explored in healthy volunteers and, in patients with Cushing’s disease and in patients receiving excessive corticosteroid replacement in conditions of adrenal insufficiency. In these situations a potent suppression of anabolic bone formation is evident, as seen by a marked decrease in circulating markers of osteoblastic bone formation, such as P1NP and osteocalcin (76). This reflects a wider uncoupling of formation and resorption in bone where changes in osteoclastic resorption in response to GCs are less evident or entirely absent (76–78). In patients with Cushing’s disease, GC excess increases the risk of fractures secondary to suppressed bone formation (79, 80). The direct and indirect mechanisms whereby exogenous GCs influence bone formation by osteoblasts are reviewed in greater detail elsewhere (81). This review will now examine studies that have aimed to delineate the contribution of GC metabolism by 11β-HSD1 to GIOP.

### The Role of 11β-HSD1 in Osteoblasts and Osteocytes in Glucocorticoid Excess

Several clinical studies have identified links between dysregulated bone metabolism and 11β-HSD1 activity in patients receiving therapeutic GCs (54, 67, 82). Increasing 11β-HSD1 activity and its inactive GC substrate availability were shown to correlate with decreased serum markers of bone formation, P1NP and osteocalcin. These data indicate that the pre-receptor activation of therapeutic GCs by 11β-HSD1 mediate this suppression of bone formation, either directly within the osteoblast themselves or through an alternative indirect pathway. A direct role for 11β-HSD1 within osteoblasts and osteocytes in mediating these effects is supported by evidence of significant expression and activity in these cell subsets when examined in human bone and primary cultures (67, 83). However, further insights into the mechanisms underpinning this have been limited to *in vitro* studies in primary human and murine osteoblasts cultures. In this context supra-physiological levels of corticosteroids promote osteoblast differentiation and support osteoid deposition (41–43). Whether these findings reflect a failing of these *in vitro* models or are instead evidence of an indirect mechanism whereby 11β-HSD1 indirectly regulates bone formation, such as through influencing circulating anabolic and anti-anabolic factors (such as androgens or parathyroid hormone) at alternative sites have yet to be adequately answered. Further insights have instead come from murine models of corticosterone excess. Global genetic deletion of 11β-HSD1 protects against the anti-anabolic effects of therapeutic GCs in bone (13). This is characterised by preservation of trabecular volume, serum measures of bone formation and preservation of osteoblast and osteocyte numbers following 4 weeks of GC exposure. These findings mirror observations in similar animal models of GIOP with osteoblast targeted blockade of GC signalling, supporting the concept that 11β-HSD1 directly mediates the anti-anabolic actions of GCs in bone (84). Studies utilising osteoblast targeted transgenic deletion of 11β-HSD1 are now still required to validate these findings. Regardless, these studies reveal a critical role for 11β-HSD1 in the suppression of bone formation in GIOP and provide evidence for the efficacy of therapeutic inhibitors of 11β-HSD1 in conditions of GC excess.

### The Role of 11β-HSD1 in Osteoclasts in Glucocorticoid Excess

Whilst the actions of therapeutic GCs have been shown to promote early osteoclast differentiation, and suppress bone resorption by mature osteoclast, the role of 11β-HSD1 within the osteoclast in GIOP is less well defined in the context (76–78, 85). Whilst a decrease in bone resorption markers have been reported in healthy volunteers receiving the 11β-HSD inhibitor carbenoxolone and then the inactive GC prednisolone, there has been limited evidence to support a role for 11β-HSD1 in any increases in bone resorption markers in GIOP. Whilst *in vitro* examination of the role of 11β-HSD1 in osteoclasts in GIOP are lacking, further insights are apparent from murine models examining animals with transgenic deletion of 11β-HSD1 (13). Here, oral GCs result in only a minor trend towards increased osteoclast numbers and bone resorption markers with no protection from this conferred in animals lacking 11β-HSD1. Together, these studies imply that 11β-HSD1 plays a limited role in mediating increased bone resorption in conditions of GC excess. However, these observations may be hampered by the relatively small contribution that osteoclasts play in mediating GIOP, relative to the impact on bone formation.

Collectively, these studies suggest that in conditions of endogenous and therapeutic GC excess, inhibition of 11β-HSD1 prevents the anti-anabolic actions of GCs and preserves bone mass, whilst their impact on osteoclast numbers and activity appear minimal.

## Glucocorticoid Activation by 11β-HSD1 Prevents Osteoclastogenesis and Bone Resorption in Inflammatory Disease

Systemic bone loss and increased risk of fracture at sites such as the femoral neck, trochanter and spine are hallmarks of patients with many chronic inflammatory diseases (86, 87). Here, circulating inflammatory mediators such as tumour necrosis factor α (TNFα) and interleukin-6 (IL-6) are predictors of decreased bone mineral density and increased fracture risk. Both bone formation and resorption show dysregulation in chronic inflammatory disease such as rheumatoid arthritis, with decrease in P1NP and increases in carboxy (C) terminal telopeptide of type I collagen (CTx) showing strong correlation with markers of disease activity (88, 89). At the cellular level, pro-inflammatory factors act on osteoblasts to directly suppress differentiation and osteoid deposition, whilst their actions on osteoclasts are mediated both directly to increase activity, and indirectly through the RANKL/OPG signalling pathway to increase both numbers and activity (90). The *in vitro* mechanisms underpinning these actions are diverse and the subject of numerous reviews (81, 91). Interest in the role of endogenous GC activation by 11β-HSD1 in this context have been fuelled by studies reporting marked increases in enzyme activity, both systemically and at sites of inflammation (92–96). In this context 11β-HSD1 has been shown to mediate anti-inflammatory GC signalling, supporting resolution and tissue repair. However, in the context of persistent and chronic autoimmune inflammation, its role has been hypothesised to switch to driving ongoing localised GC excess. To date, clinical studies have yet to examine the interaction between 11β-HSD1, bone metabolism and fracture risk in an inflammatory disease cohort. Similarly, clinical trials of 11β-HSD1 inhibitors have yet to examine their impact on bone metabolism in the context of inflammatory disease. To date, no aberrant observations of altered inflammatory responses or dysregulated bone metabolism have been reported in these studies (1–8). At present, significant gaps are present in our knowledge of the role of inflammatory 11β-HSD1 activity in bone cells in chronic inflammatory disease.

### The Role of 11β-HSD1 in Osteoblasts and Osteocytes in Inflammatory Disease

**Figure 2:**
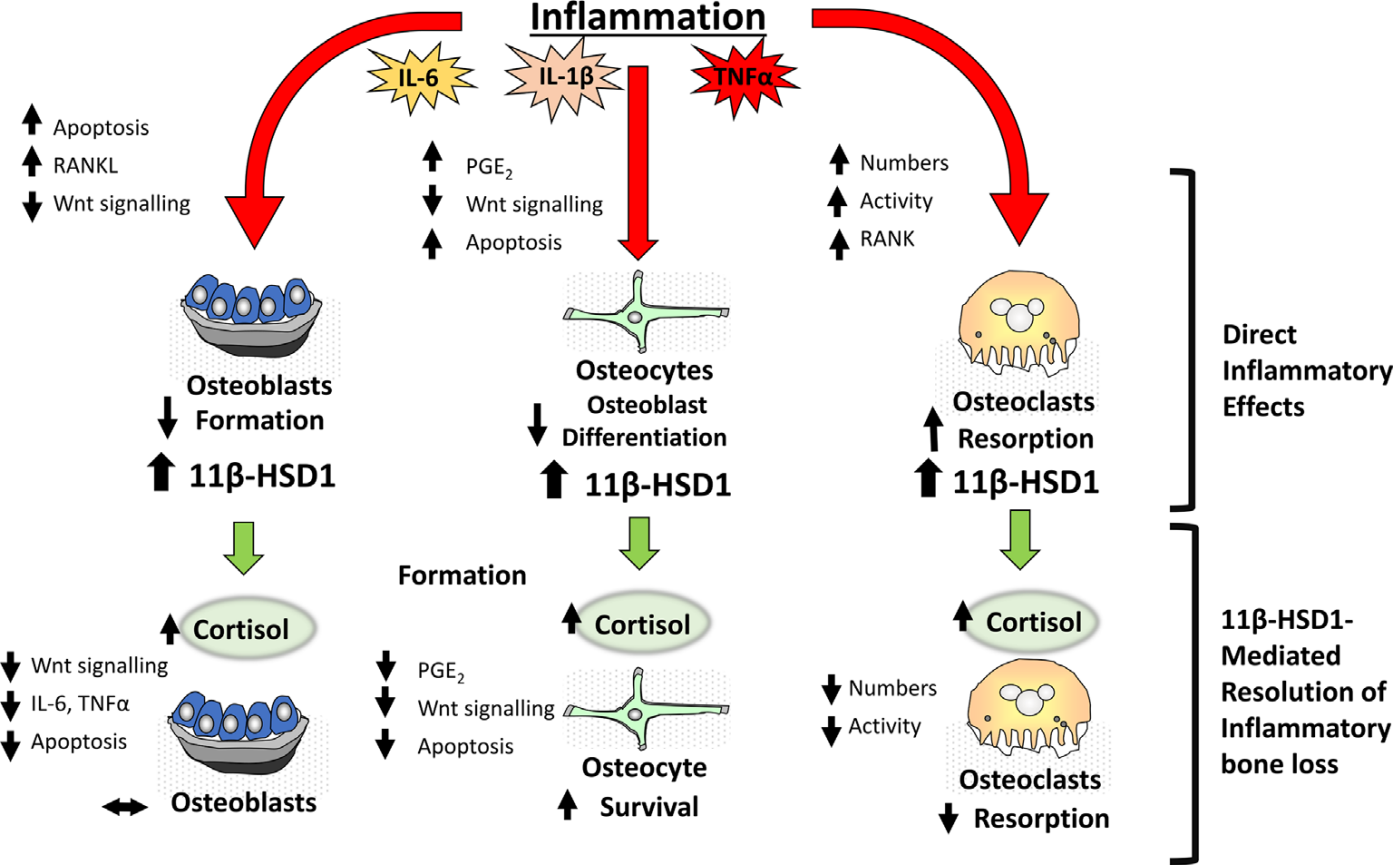
During inflammation within bone, pro-inflammatory factors including IL-6, IL-1β and TNFα have direct effects on bone cells including osteoblasts, osteocytes and osteoclasts resulting in a net loss in bone in addition to a significant induction of the corticosteroid activating enzyme 11β-HSD1. This in turn drives an anti-inflammatory resolution response where increased cortisol activation by 11β-HSD1 promotes a reduction in osteoclastic bone resorption thus suppressing inflammatory bone loss.

*In vitro* studies have revealed a potent upregulation of 11β-HSD1 in mesenchymal derived cell populations, including osteoblasts by pro-inflammatory factors such as TNFα and IL-1β (25, 83) (**Figure 2**). This inflammatory induction of 11β-HSD1 is in turn synergistically upregulated in combination with GCs, through a mechanism involving the suppression of p38-mitogen-activated protein kinase (MAPK) and upregulation of nuclear factor kappa-light-chain-enhancer of activated B cells (NFκB) signalling (25). Together, this leads to a potent increase in local GC activation in osteoblasts in response to inflammation within bone, where they then suppress local inflammatory mediators in a feedback mechanism that appears to support resolution of inflammation (83). The impact of this synergistic inflammatory regulation on bone metabolism in chronic inflammatory diseases such as rheumatoid arthritis is less clear. *In vitro* experiments using human primary osteoblast cultures reveal that these endogenous GCs can suppress osteoblast maturation and collagen deposition. *In vivo* rodent models of polyarthritis offer further insights into these processes. Here, global deletion of 11β-HSD1 severely exacerbates systemic bone loss, and suppresses anabolic bone formation, with a marked reduction in markers of mature osteoblasts, including runt-related transcription factor 2 (Runx2) and OPG (26). These findings would suggest that the anti-inflammatory actions of local GC activation by 11β-HSD1 in this context outweigh their detrimental anti-anabolic actions in osteoblasts. However, targeted mesenchymal genetic deletion of 11β-HSD1 (including in osteoblasts), failed to reproduce this systemic bone loss phenotype, indicating that the expression of 11β-HSD1 in osteoblasts may not play a critical role in mediating this inflammatory bone loss phenotype. More targeted studies, examining osteoblast specific deletion of 11β-HSD1, are now required to further explore these findings. This is particularly since global deletion of 11β-HSD1 exacerbates the severity of systemic inflammation, which is itself a confounder mediating increased bone loss. Overall, these studies point to an important role for 11β-HSD1 in regulating bone formation in chronic inflammatory diseases and bone inflammation.

### The Role of 11β-HSD1 in Osteoclasts in Inflammatory Disease

Whilst attenuated bone formation undoubtedly plays a role in abnormal bone metabolism in systemic inflammatory diseases, such as polyarthritis, a marked increase in osteoclastic bone resorption remains the primary mediator of the rapid bone loss observed in this context (90). Whilst osteoclasts, and their myeloid precursors express 11β-HSD1, its inflammatory regulation *in vivo* is less well characterised (67). Numerous studies examining myeloid precursors demonstrate a robust upregulation of 11β-HSD1 over differentiation and in response to inflammatory mediators such as TNFα, whilst similar studies in osteoclasts are lacking (97, 98). However, murine models have significantly advanced our understanding of the contribution of 11β-HSD1 in osteoclasts in this setting. In murine models of polyarthritis, osteoclast mediated bone loss is markedly exacerbated in animals with global deletion of 11β-HSD1, where it is the overriding factor driving inflammatory bone loss through a shift in RANKL/OPG signalling (9, 26, 99). These studies reveal a critical role for 11β-HSD1 in protecting against inflammatory bone resorption and supporting resolution and repair in bone (**Figure 2**). As in osteoblasts, whether these actions are mediated directly by 11β-HSD1 expression within the osteoclast, or indirectly through altered expression of local or systemic inflammatory mediators that drive this increase in osteoclast mediated bone resorption has yet to be elucidated and can only be addressed with osteoclast targeted models of 11β-HSD1 deletion.

Together, these studies indicate that systemic inflammation in conditions such as polyarthritis, 11β-HSD1 inhibition appears to have limited affects in osteoblasts, but significantly exacerbates inflammatory mediated osteoclast bone resorption and promotes systemic bone loss.

## 11β-HSD1 Protects Against Inflammation-Induced Bone Resorption in Response to Therapeutic Glucocorticoids

**Figure 3:**
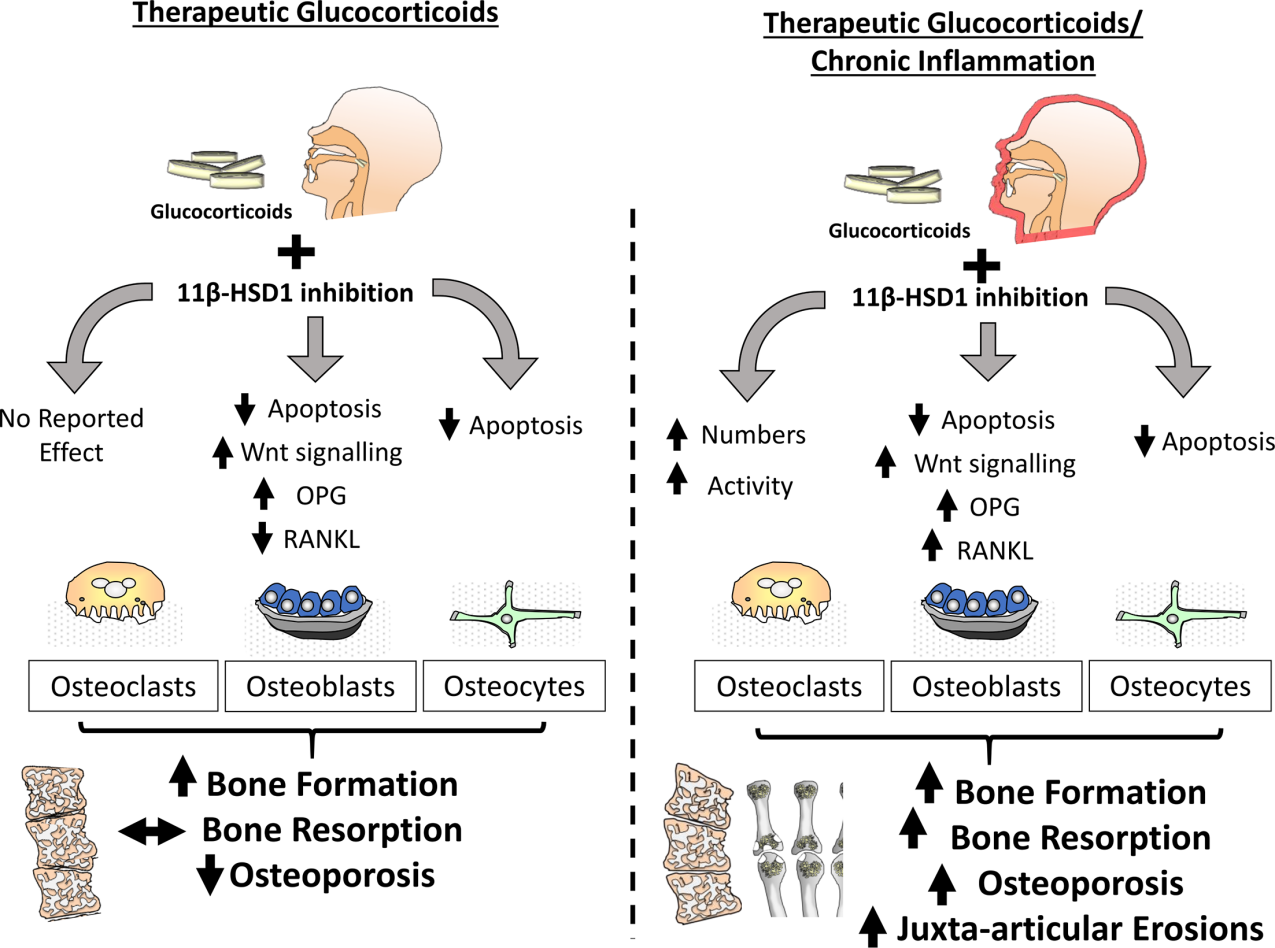
In the absence of inflammation within bone, inhibition of 11β-HSD1 protects against the actions of exogenous glucocorticoids in both osteoblasts and osteoclasts. The effect of these inhibitors is characterised by a protection from glucocorticoid induced apoptosis in both osteoblasts and osteocytes, preventing a glucocorticoid suppression of bone formation. During chronic inflammatory diseases such as rheumatoid arthritis, the protective actions of 11β-HSD1 inhibition in response to exogenous therapeutic glucocorticoids in osteoblasts and osteocytes remains evident but is overshadowed by a resistance to the anti-inflammatory properties of exogenous glucocorticoids that results in aberrant osteoclast numbers and activation. Consequently, 11β-HSD1 inhibition results in rapid bone resorption and exacerbation of both local and systemic inflammatory bone loss.

In regard to bone metabolism, the effect of therapeutic GCs in both acute and chronic inflammatory disease settings remains of significant interest. Understanding the opposing actions of GCs on bone metabolism in systemic inflammatory diseases, such as rheumatoid arthritis, where they suppress the inflammatory mediators that drive bone loss, whilst also acting directly on bone cells to drive GC-induced bone loss, remains paramount. Clinical studies examining this facet of GC action in chronic inflammatory disease are limited by confounding factors such as concurrent anti-inflammatory therapies, patient variation and disease pathophysiology, and differences in steroid dose and duration. Therefore, it is perhaps unsurprising that responses to GCs in this context report divergent outcomes, including both improvements in and worsened bone outcomes in patients with inflammatory disease (100–103). Murine models of chronic inflammatory arthritis receiving therapeutic GCs have examined this process under more controlled experimental conditions and revealed that GCs play an important role in protecting against acute inflammatory bone loss mediated by osteoclastic bone resorption, with both osteoclast numbers and activity being significantly reduced (99) (**Figure 3**). Anabolic bone formation was also significantly suppressed with GC administration, however its actions on total bone metabolism were subtle relative to the rapid inflammatory osteoclast mediated bone loss. Whilst studies examining the contribution of 11β-HSD1 to these findings are limited, several *ex vivo* studies provide insight into this process.

### The Role of 11β-HSD1 in Osteoblasts and Osteocytes in Response to Therapeutic GCs in Inflammatory Disease

Whilst therapeutic GCs suppress bone formation by osteoblasts, in models of chronic inflammatory polyarthritis, the relative contribution of 11β-HSD1 to these phenotypes are yet to be reported (9, 99). Animal models of chronic inflammatory disease with both global and osteoblast targeted transgenic deletion of 11β-HSD1 are required to assess impact of therapeutic corticosteroids in this setting. Given the potent suppression of bone formation by therapeutic GCs, and the efficacy of transgenic deletion of 11β-HSD1 in preventing this, it could be predicted that inhibition of 11β-HSD1 in patients with chronic inflammation and receiving therapeutic GCs might have a similar protection from their anti-anabolic actions in osteoblasts and osteocytes (99). However, considering the exacerbation of inflammation in response to 11β-HSD1 deletion it may be that the inflammatory suppression of bone formation is increased and coupled with a shift towards increased pro-inflammatory factors by osteoblasts, such as RANKL, TNFα and IL-6 by osteoblasts that would favour osteoclast mediated bone resorption and bone loss (9, 26, 104, 105). Further considerations should include the lesser role of dysregulated bone formation in acute inflammatory bone loss where osteoclast mediated bone resorption has been shown to play a greater role (106–108). Consequently, it may be that any beneficial effects of 11β-HSD1 inhibition on bone formation in the context of inflammatory disease may be limited or realised over longer periods of GC administration (**Figure 3**).

### The Role of 11β-HSD1 in Osteoclasts in Response to Therapeutic GCs in Inflammatory Disease

Increased bone resorption by osteoclasts is recognised as the primary driver of bone loss in many inflammatory diseases and this is rapidly suppressed in response to therapeutic GCs, however no clinical or *in vitro* studies have examined the role of 11β-HSD1 in this setting (106–108). Insights into this facet of GC action have instead come from a single study examining *in vivo* models of chronic inflammatory polyarthritis and therapeutic GC administration in animals with global and myeloid targeted transgenic deletion of 11β-HSD1 (9). In this study, suppression of osteoclast bone resorption by oral GCs was almost entirely abrogated in animals with global and myeloid targeted deletion of 11β-HSD1, revealing a critical role for local GC activation by this enzyme in suppressing osteoclast numbers and activity in chronic inflammation. Whether these effects reflect a direct autocrine effect of GC activation by 11β-HSD1 within the osteoclast, or instead reflect wider changes in pro-inflammatory factors such as RANKL, TNFα and IL-6 that drive osteoclast driven bone resorption has yet to be determined (26, 104, 105). 11β-HSD1 has been shown to influence the RANKL/OPG ratio in murine models of inflammation and this could play a role in regulating inflammatory bone resorption (81). To validate these findings now requires more targeted Cre driven deletion of 11β-HSD1 in the osteoclast subset and *in vitro* experiments that can delineate inflammatory regulation and functional consequences of autocrine GC activation by 11β-HSD1 within osteoclasts. Despite the need for future work, it is clear that 11β-HSD1 is a critical mediator in suppressing bone resorption in response to GCs and mediating their rapid bone protective actions in this context.

Together, these studies reveal that in the context chronic inflammatory diseases, such as rheumatoid arthritis, whilst 11β-HSD1 inhibition prevents the anti-anabolic actions of therapeutic glucocorticoids in osteoblasts, they abrogate GC mediated suppression of osteoclast activity and inflammatory bone loss.

## Potential Roles for 11β-HSD1 in Bone Lining Cells and Endothelium

The endothelial cells that form the vascular structures of the skeletal system are increasingly seen as important in the processes of bone formation and maintenance. These vessels supply vital nutrients and signalling molecules to osteoblasts and osteocytes, which in turn act on endothelial cells to support further vascular development, hence the processes of angiogenesis and osteogenesis are said to be “coupled” (109). Osteoblasts and their progenitors are therefore found in close proximity to these osteogenesis-promoting endothelial cells, termed type H vessels due to their high expression of adhesion molecule CD31 and the endothelial sialomucin endomucin (110, 111). Osteoblasts and osteocytes support angiogenesis by producing vascular endothelial growth factor (VEGF) *via* the hypoxia-inducible factor α (HIFα) pathway (112, 113). VEGF stimulates blood vessel invasion by acting on endothelial cells, but also promotes migration and activation of osteoblasts, linking these complementary functions of angiogenesis and osteogenesis in formation or remodelling of bone (114, 115). Similarly, preosteoclasts secrete platelet-derived growth factor-BB (PDGF-BB) which promotes angiogenesis by recruiting endothelial and mesenchymal progenitors and inducing formation of type H vessels, this in turn stimulates bone formation and remodelling (116, 117). These processes are all attenuated by GC treatment, as seen in both *in vitro* and *in vivo* models (118–120). It is not known what role, if any, 11β-HSD1 plays in type H vessel cells. However, expression of 11β-HSD1 has been identified in vascular endothelial cells and found to inhibit angiogenesis by interfering with endothelial cell morphological changes required for tube formation (121–124).

Bone lining cells (BLCs) are derived from mature quiescent osteoblasts and thought to perform a number of functions in skeletal homeostasis [reviewed in detail by Wein (125)]. It is not known whether BLCs express 11β-HSD1 like other mesenchymal derived cells, if so it may perform a similar function as in osteoblasts under conditions of inflammation, ageing and GC excess (25). Treatment of mice with prednisolone was found to inhibit BLC activation and proliferation, including their conversion into new osteoblasts (126). However, despite their importance in bone growth and repair, much remains to be elucidated about the functions of BLCs and the importance of 11β-HSD1 metabolised GCs in this cell type.

## Final Conclusions

The studies highlighted in this review reveal a complex role for 11β-HSD1 in bone remodelling, with some limited evidence for a role in normal physiology, but a much greater role in mediating the actions of GCs in conditions of exogenous and endogenous corticosteroid excess and inflammation. With the interest in therapeutic inhibitors of 11β-HSD1, these studies point to the potential for their application in conditions such as Cushing’s disease, where they would be predicted to prevent the anti-anabolic action of GCs on bone to reduce the risks of osteoporosis and fracture. In contrast, their application in the context of inflammatory disease appears to be complicated by the risk of exacerbating inflammatory bone loss by osteoclasts. Therefore, in inflammatory disease the role of 11β-HSD1 appears to be protective, mediating the suppression of inflammatory factors that drive bone resorption and decrease osteoclast numbers and activity. Rather than inhibiting 11β-HSD1, approaches may instead benefit from targeting therapeutic GCs selectively to leukocyte and osteoclast populations to more effectively deliver their beneficial bone sparing actions in the context of inflammation. Whether the inflammatory induction of 11β-HSD1 within bone resorbing cells could be used to selectively facilitate metabolic targeting of GCs for intracellular activation within osteoclasts to suppress inflammatory bone resorption without driving off-target metabolic side effects has yet to be determined.

## Author Contributions

RH and MC devised topic. MC, RH, and CM jointly wrote manuscript and devised figures. All authors contributed to the article and approved the submitted version.

## Funding

This research was supported by the Wellcome Trust (reference: 215243/Z/19/Z).

## Conflict of Interest

The authors declare that the research was conducted in the absence of any commercial or financial relationships that could be construed as a potential conflict of interest.

## Publisher’s Note

All claims expressed in this article are solely those of the authors and do not necessarily represent those of their affiliated organizations, or those of the publisher, the editors and the reviewers. Any product that may be evaluated in this article, or claim that may be made by its manufacturer, is not guaranteed or endorsed by the publisher.

## Abbreviation

11β-HSD1, 11β-hydroxysteroid dehydrogenase type 1; 11β-HSD2, 11β-hydroxysteroid dehydrogenase type 2; BLCs, bone lining cells; CBG, corticosteroid-binding globulin; CTx, carboxy terminal telopeptide of type I collagen; DXA, dual-energy X-ray absorptiometry; GCs, glucocorticoids; GIOP, glucocorticoid induced osteoporosis; GR, glucocorticoid receptor; HIFα, hypoxia-inducible factor alpha; HPA, hypothalamic-pituitary-adrenal; IL-6, interleukin 6; MAPK, mitogen-activated protein kinase; NAD, nicotinamide adenine dinucleotide; NADPH, nicotinamide adenine dinucleotide phosphate; NFκB, nuclear factor kappa-light-chain-enhancer of activated B cells; OPG, osteoprotegerin; P1CP, procollagen type 1 carboxy-terminal propeptide; P1NP, procollagen type 1 amino-terminal propeptide; PDGF-BB, platelet-derived growth factor-BB; RANK, receptor activator of nuclear factor kappa-B; RANKL, receptor activator of nuclear factor kappa-B ligand; Runx2, runt-related transcription factor 2; THE, tetrahydrocortisone; THF, tetrahydrocortisol; TNFα, tumour necrosis factor alpha; VEGF, vascular endothelial growth factor.
